# CDK12 Promotes Cervical Cancer Progression through Enhancing Macrophage Infiltration

**DOI:** 10.1155/2021/6645885

**Published:** 2021-02-11

**Authors:** Bikang Yang, Jing Chen, Yincheng Teng

**Affiliations:** Department of Gynecology and Obstetrics, Shanghai Jiao Tong University Affiliated Sixth People's Hospital, China

## Abstract

Cervical cancer (CC) is a commonly diagnosed and primary consideration of cancer patient death in female reproductive system malignancy. Cyclin-dependent kinase 12 (CDK12), as a transcription-associated CDK, plays important roles in tumor-promoting behaviors, whereas the underlying mechanisms of *CDK12* in CC progression are still obscure. In this report, we investigated the role of *CDK12* in cervical cancer. The current study identified *CDK12* mRNA and protein expression remarkably upregulated in CC patients. Upregulated *CDK12* was closely associated with CC progression and poor prognosis. *In vitro* and *in vivo* functional experiments showed that knockdown of *CDK12* inhibited cancer cell proliferation and colony formation and promoted apoptosis. Further investigations demonstrated that *CDK12* regulated the immune microenvironment to facilitate the progression of CC cells by promoting macrophage infiltration. Meanwhile, we first demonstrated that nuclear import of CDK12 is mediated by TNPO1 and might be a new therapeutic target in oncology. Collectively, this study pointed out the potential of *CDK12* to serve as a novel therapeutic target in restricting CC proliferation and cell cycle process through promoting macrophage infiltration.

## 1. Introduction

The incidence of cervical cancer (CC) is becoming one of the most common female reproductive system malignancies, especially in lower HDI (Human Development Index) settings, despite progress in prophylactic vaccines [1]. Meanwhile, early cervical cancer can be treated with surgery or radiation, but uptake remains poor in advanced stage CC [2]. Hence, deeper comprehension of the molecular mechanism of CC aggression will be critical for the development of effective strategies for CC treatment.

Cyclin-dependent kinases (CDKs) are the important regulators of the cell-cycle biological process [3]. CDKs are usually divided into two subfamilies: such as CDK2 and CDK4, which directly regulate cell-cycle progression. Other CDKs are related to gene transcription, such as CDK12 and CDK13, which mainly are involved in posttranscriptional mRNA processing [4]. A mass of study pieces of evidence revealed the downregulation or upregulation of CDKs in a diverse range of cancers [5]. In this context, malfunction of several transcription-associated CDKs has been linked to tumorigenesis and progression, supporting that posttranscriptional mRNA processing plausible correlations with cancer etiopathogenesis [6]. Therefore, CDKs have been tested extensively as potential targets for cancer therapy.

Cyclin-dependent kinase 12 (CDK12), as a transcription-associated CDK, complexes with cyclin K to modulate gene transcription elongation through phosphorylation of the RNA polymerase II (RNA Pol II) C-terminal domain (CTD) at Ser2 [7]. *In vitro*, CDK12 also potentially modulates transcription initiation by phosphorylation of RNA Pol II at Ser5 and takes part in a series of cellular biological processes including DNA damage response (DDR), the proliferation of the cell, splicing of precursor mRNA, and the process of pretranscriptional mRNA [8, 9]. Moreover, CDK12 also executes essential missions in cell-cycle process regulation. The previous studies have found that there were high-frequency mutation and amplification expression of CDK12 in several malignancies, including ovarian, breast, and prostate cancers [10, 11]. Accordant with its function in the maintenance of genomic stability and cell-cycle regulation, the loss or overexpression of CDK12 is associated with the progression and metastasis of cancers [12].

The tumor microenvironment (TME), as a complex ecosystem, contains mesenchymal, neoplastic tissue, and inflammatory cells and contributes to cancer cell growth, invasion, and metastasis [13]. Inflammatory cells as a major component of the TME, among which tumor-associated macrophages (TAMs), play an important role in tumorigenesis and progression [14]. TAMs promote tumor cell survival and proliferation by releasing platelet-derived growth factor proinflammatory (PDGF), stimulate tumor angiogenesis by releasing vascular endothelial growth factor (VEGF), and contribute to tumor metastasis by inducing the nuclear factor-kappa B (NF-*κ*B) pathway [15]. Therefore, targeting TAMs has tremendous feasibility to become a promising and efficient therapeutic strategy by combination with traditional therapy and has attracted comprehensive attention in targeting TAMs for cancer immunotherapy, which is aimed at the body's immunocompetence [16].

In the present study, the expression of *CDK12* was remarkably elevated in cervical cancer compared with normal cervical and tended to have a poor prognosis. The knockdown of *CDK12* inhibited CC cell proliferation, induced cell cycle arrest at G2/M, and a higher ratio of CC cell apoptosis. Further investigations demonstrated that CDK12 promoted macrophage infiltration and regulated the immune microenvironment in cervical cancer cells. Moreover, our results revealed a new molecular mechanism that nuclear import of CDK12 is mediated by TNPO1 and might be a new therapeutic target in oncology.

## 2. Method

### 2.1. Human CC Tissue Samples

In our study, freshly frozen tissues, which obtain 20 CC tissue specimens and 20 cervical epithelial tissue specimens, were collected from the Shanghai Sixth Hospital. All patients were confirmed CC by pathologist diagnosis. None of the patients underwent chemoradiotherapy before operative treatment. This research was authorized by the Shanghai Municipal Government, and informed consent was provided to all patients before this study.

### 2.2. Cell Culture

Human cervical cancer HeLa and Siha cells were preserved in Shanghai Cancer Institute, Ren Ji Hospital, School of Medicine, Shanghai Jiao Tong University. HeLa and Siha cells were maintained in DMEM (Gibco) and supplemented with 10% fetal bovine serum and (v/v) pen/strep antibiotics. All of them were incubated at 37°C and atmosphere containing 5% CO_2_.

### 2.3. Immunohistochemical Staining

CC tissue, normal cervical epithelial tissue, and mice xenograft tissue samples were bedded in paraffin for immunohistochemistry. IHC staining was shown as previous research [12]. The primary antibody used was anti-CDK12 (dilution 1 : 1000, 11973s, CST). In the last step, all the specimens were examined and taken pictures with a microscope. The staining intensity was scored as follows: appreciable staining scored 0, lesser brown staining scored 1, brown staining scored 2, and dark brown scored 3, and the extent of staining was scored as 0 (<10%), 1 (10–40%), 2 (41–70%), or 3 (>70%). Samples with a final score of no more than 3 were defined as “low expression.” These scores were assessed independently by two pathologists in a blinded manner.

### 2.4. Quantitative Real-Time PCR

Total mRNA was isolated from CC cells using TRIzol reagent (TaKaRa) following the operating specifications. Quantitative real-time PCR was performed with SYBR Premix Ex Taq (TaKaRa) on a 7500 Real-Time PCR system (Applied Biosystems, Inc. USA). The relative mRNA levels of each gene were normalized to the expression of the reference gene 18S. The primer sequences used for *CDK12* and 18s RNA detection were as follows:

*CDK12* forward 5′-CTAACAGCAGAGAGCGTCACC-3′,

*CDK12* reverse 5′-AAAGGTTTGATAACTGTGCCCA-3′;

18s forward 5′-TGCGAGTACTCAACACCAACA-3′,

18s reverse 5′-GCATATCTTCGGCCCACA-3′;

The formula RQ = 2 − ΔCt was used to quantify the relative target gene expression levels for statistical analysis.

### 2.5. Short Hairpin RNA Mediated Gene Knockdown

shRNAs targeting different genes were purchased from Gene Pharma (Shanghai, China). Cancer cells were transduced with 1.5 × 10^6^ recombinant lentivirus-transducing units in the presence of 5 *μ*g/mL Polybrene (H9268, Sigma). For lentiviral vector-related work, cells (48 h posttransduction) were selected and maintained in 2 *μ*g/mL puromycin. The efficiency of the knockdown was tested by quantitative real-time PCR and western blot. The clone IDs for the CDK12 shRNA were:

sh1,5′-GATCCGCCTTCAAACTAGACCGAAGGTTCAAGAGACCTTCGGTCTAGTTTGAAGGCTTTTTTG-3′,

sh2,5′GATCCGGAGAAGACCAGGAAAGAACGTTCAAGAGACGTTCTTTCCTGGTCTTCTCCTTTTTTG-3′.

### 2.6. Western Blotting

Whole-cell protein lysates were made according to routine protocols, and western blotting was performed as previously described [13]. The antibodies used were anti-CDK12 (11973s, CST), anti-GAPDH (60004-1-Ig, Proteintech), and anti-TNPO1 (ab10303, Abcam). For immunoprecipitation, extracts were incubated with protein A/G Dynabeads (Bimake). After that, beads were washed three times with phosphate-buffered saline, resuspended in 1x protein SDS loading, and boiled for 15 mins. Subcellular nuclear cytoplasm protein fractionation was performed using the NE-PER Nuclear and Cytoplasm Extraction Reagents (Thermo Fisher Scientific) following the protocol supplied by the manufacturer. Secondary antibodies were used at 1 : 5000, including antimouse (G-21040, Invitrogen) and antirabbit (G-21234, Invitrogen), respectively.

### 2.7. Cell Viability Assay

HeLa and Siha cells were seeded into 96-well plates with 200 *μ*L medium (2000 cells/well) and cultured at 37°C. After adding 10% Cell Counting Kit-8 reagent (CCK-8, Dojindo), respectively, the cells were cultured at 37°C for 1 hour. The cell viability was measured at 450 nm with a Power Wave XS microplate reader (BioTek, Winooski).

### 2.8. Colony Formation Assay

After cell transfection with sh-*CDK12*-1, sh-*CDK12*-2, or shNC, HeLa and Siha and their control cells were plated in 6-well plates (1.5 × 10^3^ cells). After 10-14 days, colonies were washed by PBS and stained with 0.05% crystal violet for 15 min. The number of visible colonies was counted under microscopy.

### 2.9. Cell Apoptosis Flow Cytometry Assay

HeLa and Siha cells, which were transfected with sh-*CDK12*-1, sh-*CDK12*-2, or shNC, were cultured under serum deprivation for 24 hours to assess apoptosis. Using 0.25% trypsin, cells were resuspended (100 *μ*L) for Annexin V staining with propidium iodide (PI) and Annexin V-FITC (BD Pharmingen, USA). The percentage of cells by the LSRFortessa cell analyzer (BD Biosciences) and results were calculated with FlowJo 10.0 software.

### 2.10. Cell Cycle Assay

HeLa and Siha cells, which were transfected with sh-*CDK12*-1, sh-*CDK12*-2, or shNC, were cultured under serum deprivation for 48 hours to assess the cell cycle. The cells were fixed in 75% ice ethyl alcohol at 4°C for 12 h. After that, the cells were suspended in PBS containing 10 mg/L RNase A and 50 *μ*g/mL PI and incubated in darkness at room temperature for 30 min. The DNA content was examined by the LSRFortessa cell analyzer (BD Biosciences), and results were calculated with the Modfit LT 5.1 software.

### 2.11. Mice Xenograft Model

To generate a xenograft model, a total of 5 × 10^6^ HeLa and Siha cells transfected with sh-*CDK12*-1 or shNC in 150 *μ*l PBS were injected subcutaneously in the groin of each male BALB/c mice (5-6 weeks of age). Tumor volume was measured by Vernier caliper every 6 days. Mice were killed 28 days after injection, and the xenograft tumors were excised to measure volume and weight. All animals received humane care according to the local or national requirements for the care and use of laboratory animals.

### 2.12. Edu Stain Immunofluorescence and Confocal Microscopic Imaging

HeLa and Siha cells, which are transfected with sh-*CDK12*-1, sh-*CDK12*-2, or shNC, were seeded onto 8-well chamber slides, then incubated with serum-free media containing the indicated doses of geraniin for 24 hours and cotreated with 100 *μ*L of EdU (50 *μ*mol/L) for 2 hours. Cells were fixed with 4% paraformaldehyde at 37°C for 15 min and washed with PBS, then permeabilized with 0.5% Triton X-100. Fluorescence images were obtained using a confocal microscope (Carl Zeiss, Germany) according to the manufacturer's guidance.

### 2.13. Statistical Analysis

Data were presented as the mean ± SD. The SPSS software program (version 19.0; IBM Corporation) and GraphPad Prism 7 (La Jolla, CA) software was employed for statistical analysis. The Student *t*-test was employed to analyze two groups of data. One-way ANOVA was used for comprising of multiple groups. Values of *P* < 0.05 were considered statistically significant.

## 3. Results

### 3.1. CDK12 Contributes to the Malignant Transformation and Poor Prognosis in Cervical Cancer

**Figure 1:**
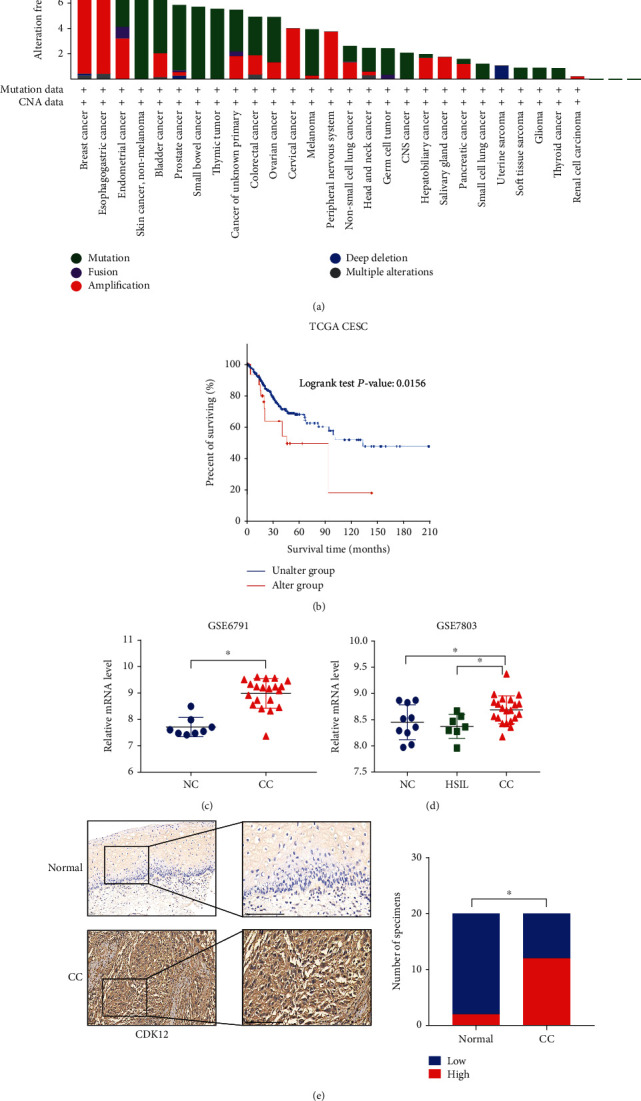
Cyclin-dependent kinase 12 (*CDK12*) overexpression and its correlation with a poor prognosis in cervical cancer (CC). (a) Analysis of *CDK12* genetic alterations across various human cancers using TCGA databases. (b) Kaplan-Meier analysis (log-rank test) of the overall survival of patients with CC based on *CDK12* genetic alteration, using TCGA databases. (c) Expression profiles of *CDK12* in tumors (T) and normal (N) cervical tissue samples GSE6791, which are from Gene Expression Omnibus datasets. (d) Expression profiles of *CDK12* in precancerous lesions of the uterine cervix, NC and CC samples from GSE7803. NC: normal cervix; HSIL: high-grade squamous intraepithelial lesion. (e) Representative immunohistochemical images and quantification analysis showing CDK12 expression in human cervical cancer and normal uterine cervix tissue specimens from Sixth hospital (sixth cohort). Scale bar: 200 *μ*m. Two-tailed *t*-test, ^∗^*P* < 0.05.

The gene mutation and amplification of *CDK12* were found in different types of malignancies by analyzing The Cancer Genome Atlas (TCGA) databases, such as amplification of *CDK12* in cervical cancer (CC), which suggested that CDK12 had some properties of an oncogene (Figure 1(a)). Of note, amplification of *CDK12* expression has shown a poor prognosis in CC patients (Figure 1(b)). To further unveil the *CDK12* dysregulation involved in the progression of CC, we initially interrogated gene expression profiles of *CDK12* in different Gene Expression Omnibus (GEO) datasets (GSE6791 and GSE7803), which all contain normal uterine cervix and CC tissues. The analysis results revealed that *CDK12* mRNA expression was remarkably increased in CC (Figures 1(c) and 1(d)). Moreover, CIN (cervical intraepithelial neoplasia) is a notable precancerous lesion of CC, especially HSIL (high-grade squamous intraepithelial lesion). Intriguingly, we indicated that the *CDK12* expression was remarkably increased in CC compared with HSIL in GSE7803 (Figure 1(d)). To evaluate the protein level of CDK12 in CC tissue specimens, we performed immunohistochemical staining in a series of patient specimens, which obtained 20 cases of CC tissue specimens and 20 cases of normal cervix tissue specimens. The analysis results indicated that the level of CDK12 protein was remarkably increased in CC compared to normal cervix tissue specimens (Figure 1(e)). Taken together, we speculated *CDK12* as the specific oncogene selectively contributing to the progression of CC.

### 3.2. CDK12 Regulates Tumorigenesis and Proliferation In Vitro

**Figure 2:**
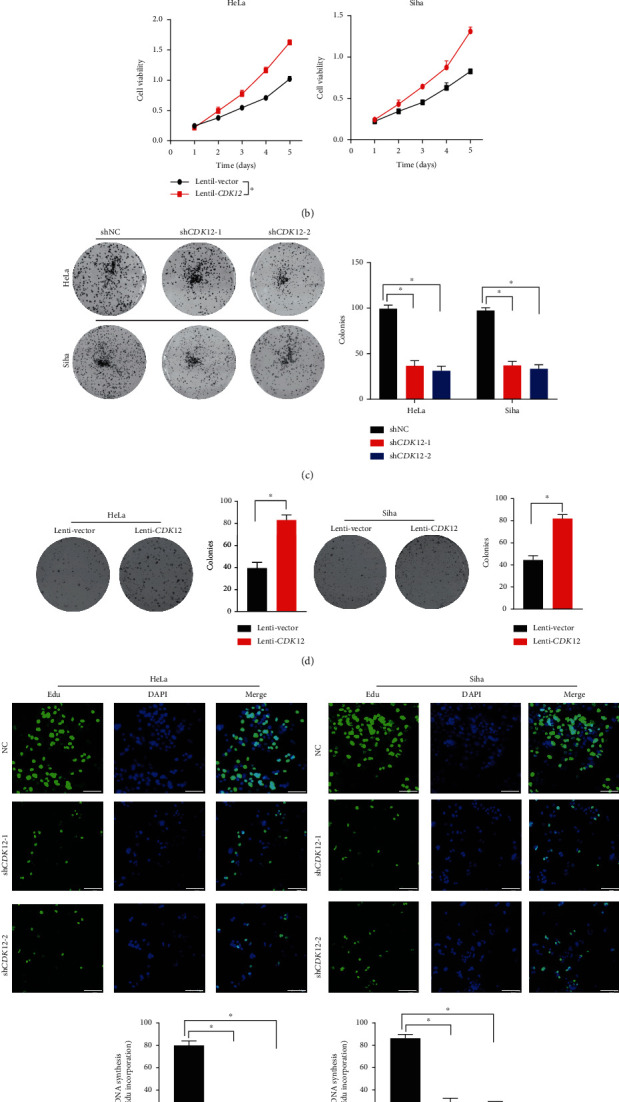
Cyclin-dependent kinase 12 (*CDK12*) promotes cervical cancer cell proliferation *in vitro*. (a) The cell growth of HeLa and Siha cells in three groups (shNC, sh*CDK12*-1, sh*CDK12*-2) were analyzed with CCK-8 assay. (b) The cell growth of HeLa and Siha cells in the two groups (lentivector and lenti-*CDK12*) were analyzed with CCK-8 assay. The results are shown as the means ± standard deviation of the OD450 value. (c) The colony formation assay and quantification of HeLa and Siha cells in three groups (shNC, sh*CDK12*-1, sh*CDK12*-2) were shown. (d) The colony formation assay and quantification of HeLa and Siha cells in two groups (lentivector and lenti-*CDK12*) were analyzed. (e) EdU staining assay analysis. Representative EdU staining and quantification ratio of proliferation cells. Error bars represent mean ± standard error of the mean. Scar bar: 50 *μ*m. Two-tailed *t*-test, ^∗^*P* < 0.05.

To elucidate the functions of *CDK12* in the progression of CC, we first selected HeLa and Siha CC cell lines, which with relatively higher expression levels of *CDK12* (supplement Figure 1(a)). We then established the knockdown of *CDK12* cells by short hairpin RNA (shRNA) targeting *CDK12*. Meanwhile, we also established the overexpression of *CDK12* cells by lentivirus-mediated transfection. Quantitative real-time PCR and western blotting confirmed the efficiency of the CDK12 knockdown and overexpression in Hela and Siha cells (supplement Figure 1(b, c)). In the presence of 10% FBS, the analysis indicated that the knock-down of *CDK12* remarkably reduced cell viability in HeLa and Siha cells, compared with shNC (Figure 2(a)). Following these findings, the overexpression of *CDK12* remarkably promoted cell viability in HeLa and Siha cells (Figure 2(b)). To further verify the role of *CDK12* in CC cell proliferation, we performed a colony formation assay. In line with these findings, the colony formation was suppressed by knockdown of *CDK12* and promoted by overexpression of *CDK12* (Figures 2(c) and 2(d)). Moreover, we performed an EdU stain assay to detected DNA replication. Our results also showed DNA replication was suppressed by knockdown of CDK12 (Figure 2(e)). Collectively, these results demonstrated that CDK12 was profoundly implicated in promoting CC proliferation.

### 3.3. Knockdown of CDK12 Regulates the Cell Cycle and Induces Apoptosis In Vitro

**Figure 3:**
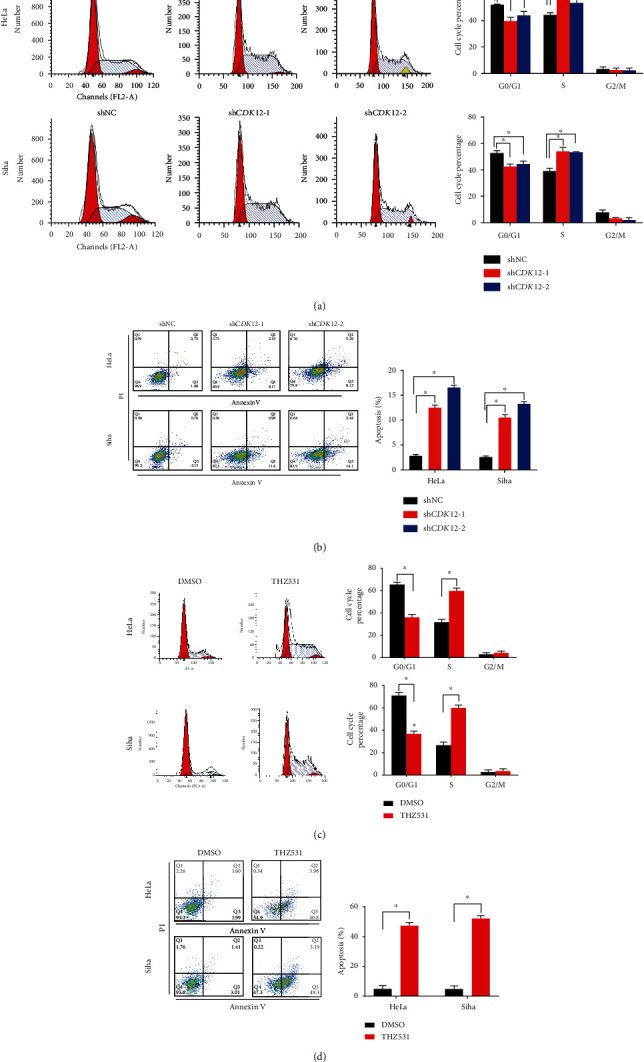
CDK12 regulates the cell cycle and protects from apoptosis in CC cells. (a) Cell cycle analysis of CDK12 knockdown versus control in HeLa and Siha cells. (b) Apoptosis analysis of CDK12 knockdown versus control in HeLa and Siha cells. (c) Cell cycle analysis of 200 nM THZ531 vs. DMSO in HeLa and Siha cells. (d) Apoptosis analysis of 200 nM THZ531 vs. DMSO in HeLa and Siha cells. Two-tailed *t*-test, ^∗^*P* < 0.05. FITC: fluorescein isothiocyanate; PI: propidium iodide.

CDK12, as an important factor regulating the cell-cycle process, is commonly involved in the growth of cancer cells. Therefore, we employed flow cytometry to examine the effect of *CDK12* on cervical cancer cell-cycle progression. FACS cell-cycle analysis results showed that knockdown of *CDK12* led to the proportion of HeLa and Siha cells in the G2/M phase and remarkably increased than the shNC group (Figure 3(a)). Moreover, we also performed annexin V (V) and propidium iodide (PI) staining and flow cytometry analysis to identify apoptotic and dead cells ratio, respectively. These analysis results showed that the apoptosis ratio of the sh*CDK12*-1 and sh*CDK12*-2 in the HeLa and Siha cell line was remarkably higher than the shNC group (Figure 3(b)). The previous discovery of the covalent CDK12 inhibitor THZ531 revealed consistently inhibited CDK12 kinase activity and suppressed cancer cell proliferation [17]. To evaluate if THZ531 performs a similar anticancer activity, we treated HeLa and Siha cells with 200 nM THZ531 for 48 hours. FACS cell-cycle analysis following treatment with THZ531 displayed a dramatic increase in the proportion of cells exhibiting G2/M content (Figure 3(c)). To investigate this further, we performed annexin V/PI staining to determine the influence of THZ531 on cervical cancer cells. At 200 nM THZ531, high doses of THZ531 treatment dramatic increase in the ratio of apoptotic cells were observed than DMSO control of the experiment (Figure 3(d)). These results indicated that the knock-down of CDK12 remarkably inhibited the cell cycle and induced apoptosis of cervical cancer cells.

### 3.4. Knockdown of CDK12 Expression Inhibits Tumorigenesis In Vivo

**Figure 4:**
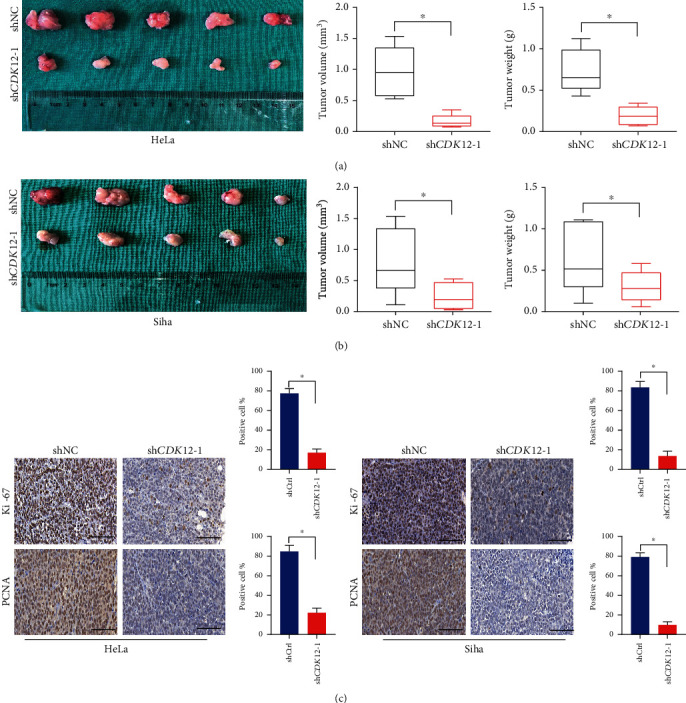
Knockdown of CDK12 suppresses tumor proliferation *in vivo*. (a), (b) HeLa and Siha cells were infected with sh*CDK12*-1 and shNC and were injected into BALB/C nude mice. Excised tumor in different groups was shown. Four weeks later, tumor volumes and weights in sh*CDK12*-1 were inhibited in comparison with shNC. (c) Typical IHC images of Ki67, and PCNA from xenografts under HeLa and Siha cells treatment with sh-NC and sh*CDK12*-1. Two-tailed *t*-test, ^∗^*P* < 0.05. Scale bar: 50 *μ*m.

To further analyze the effect of CDK12 knockdown in CC cell proliferation, *in vivo* studies were performed by subcutaneous xenograft injecting mice with sh*CDK12* in HeLa and Siha cells. The results showed that the sh*CDK12* group remarkably decreased tumor burden, as evaluated by tumor weight and tumor volume measurements in HeLa and Siha cells (Figures 4(a) and 4(b)). Furthermore, immunohistochemical staining results showed that the immunostaining intensities of PCNA and Ki-67, markers of cell proliferation, were remarkably decreased in the sh*CDK12* group compared to the corresponding shNC group in HeLa and Siha cells (Figure 4(c)). Collectively, these results revealed that the knockdown of CDK12 inhibited tumorigenesis and growth in CC.

### 3.5. CDK12 Enhances the Macrophage Infiltration to Promote CC Proliferation

**Figure 5:**
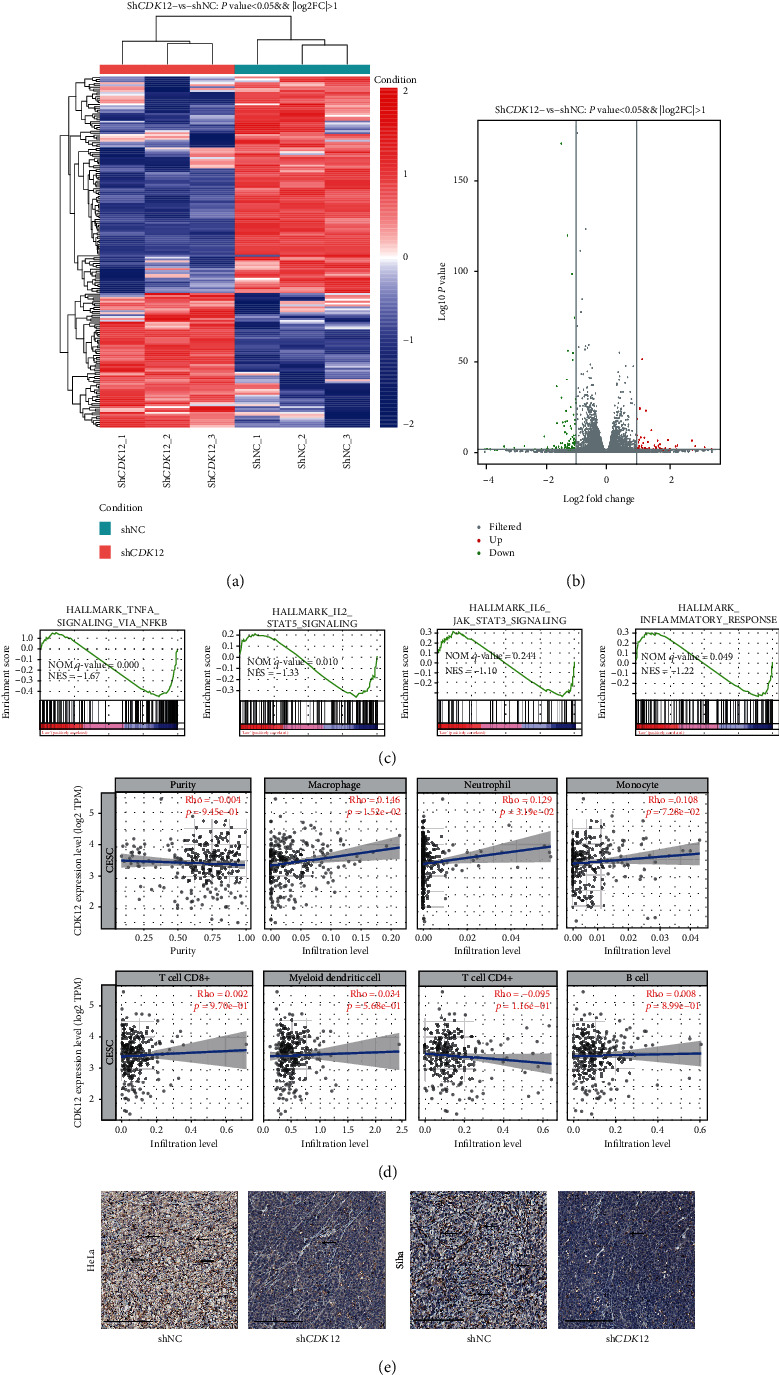
High-expression of CDK12 enhances the macrophages infiltration. (a) Heatmap of the different genes by shRNA-mediated CDK12 knockdown. (b) Volcano plotting of the different genes by shRNA-mediated CDK12 knockdown. (c) Gene set enrichment analysis (GSEA) using hallmark gene sets by shRNA-mediated CDK12 knockdown. (d) The abundances of seven inflammatory/immune infiltrates (B cells, CD4+T cells, CD8+T cells, neutrophils, macrophages, monocyte, and dendritic cells) are estimated by the TIMER algorithm. (e) Typical IHC images of F480 from xenograft under HeLa and Siha cells treatment with sh-NC and sh*CDK12*-1. Scale bar: 200 *μ*m.

Having demonstrated that *CDK12* has crucial functions in promoting CC proliferation properties, we intended to further interrogate the molecular mechanism correlation with *CDK12* function. To investigate the potential mechanism of *CDK12* in cervical cancer, we performed high throughput transcriptome sequencing (RNA-seq) analysis to compare the transcriptome for two groups: shNC and sh*CDK12* groups, respectively (Figures 5(a) and 5(b)). We performed GSEA (Gene Set Enrichment Analysis) to analyze the differentiated genes in the high and low *CDK12* expression groups in the RNA-seq database. The analysis results indicated that the high expression of CDK12 was enriched to some immune-inflammatory response pathways, such as TNFA_SIGNALING_VIA_NFKB and HALLMARK_INFLAMMATORY_RESPONSE (Figure 5(c)). *CDK12* may take part in the regulation of the immune microenvironment of CC cells. Therefore, we examined the correlation of the expression of *CDK12* and differential abundance of immune cell infiltration by using TCGA databases. The Timer Database was utilized to calculate the correlation of the expression of *CDK12* with eight types of immune cell infiltration in CC patient tissues. The analysis results showed that the expression of *CDK12* significant positive correlation with M0 macrophage infiltration (Figure 5(d)). Furthermore, immunohistochemical staining results showed that the immunostaining intensities of macrophages were remarkably decreased in the sh*CDK12* group compared to the shNC group in the subcutaneous xenograft of HeLa and Siha cells (Figure 5(e)). Taken together, these results revealed that *CDK12* promoted the proliferation of CC by regulating the immune microenvironment which depends on the enhancement of macrophage infiltration.

### 3.6. Karyopherin TNPO1 Modulates Nuclear Import of CDK12 in CC Cells

**Figure 6:**
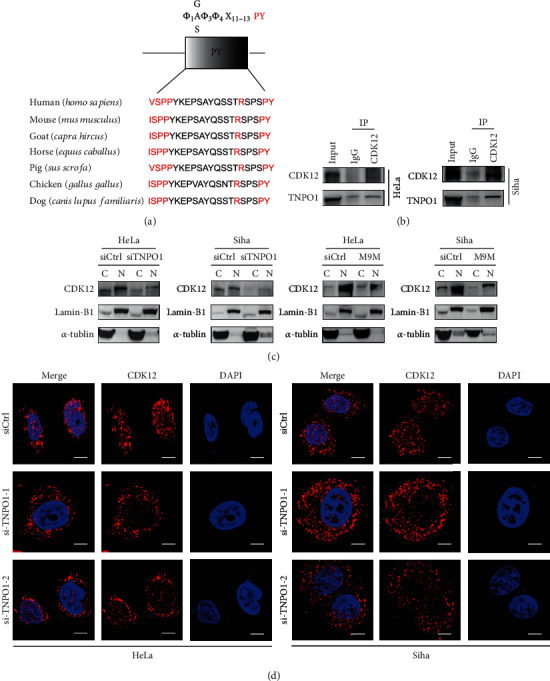
Karyopherin TNPO1 mediates nuclear import of CDK12. (a) PY-motif of the CDK12 of different species shows that the sequence of this domain is well conserved during evolution; central hydrophobic motifs and the R/K/H-PY motifs are highlighted in red. P: proline; Y: tyrosine. (b) Western blotting of TNPO1 levels following immunoprecipitation of CDK12 in HeLa and Siha cells. (c) After being transfected with siRNA targeting TNPO1 or inhibitor of the TRN pathway (M9M) vector, western blotting of CDK12 levels in the nucleoplasm and cytoplasm of HeLa and Siha cells. (d) HeLa and Siha cells were analyzed by immunofluorescence against CDK12, as labeled, during siTNPO1. DAPI staining was used to visualize nuclei. Scale bar: 20 *μ*m.

CDK12, as a serine/threonine kinase correlation with transcription, plays an important role in regulating gene expression through promoting the phosphorylation of the RNA POL II and inducing transcription elongation. To ensure accurate gene transcription-associated function of CDK12, the spatial distribution of CDK12 protein requires karyopherin*β* protein (Kap*β*s) mediation. Therefore, we further the explored potential karyopherin*β* protein (Kap*β*s) involved in the nuclear–cytoplasmic transport of CDK12. We found CDK12 at least contained a PY-NLS (proline-tyrosine amino acid) motif consensus sequence, which was recognized by Transportin 1 (*TNPO1*), by the analyzing canonical sequence of CDK12 based on the UniProt database. Then, we noted the presence of a strictly conserved arginine and downstream PY-NLS motif within CDK12 295-315 amino acid, and the motif is highly conserved in different spliceosomes of Homo sapiens and various mammalian species (Figure 6(a)). Therefore, we performed coimmunoprecipitation against CDK12 bound to TNPO1 in HeLa and Siha cells (Figure 6(b)). Meanwhile, we performed western blotting assay on separate nuclear and cytoplasmic fractions of HeLa and Siha cells and further represented that the nuclear import of CDK12 was decreased when *TNPO1* was knockdown (Figure 6(c)). Moreover, holding the particularly nuclear import of CDK12 was regulated by TNPO1, we performed a TNPO1-specific inhibitor peptide (M9M). Western blotting assay on separate nuclear and cytoplasmic fractions of HeLa and Siha cells and further represented that nuclear import of CDK12 was decreased when expressed an M9M construct (Figure 6(c)). To further verify that the nuclear import of CDK12 was mediated by TNPO1, we performed an immunofluorescence assay on these cells to further represent that CDK12 scattered localization in nucleus and cytoplasm, and nuclear localization of endogenous CDK12 was remarkably impaired when *TNPO1* was knockdown (Figure 6(d)).

## 4. Discussion

Cyclin-dependent kinases (CDKs) are important regulators of the cell-cycle process and gene transcription. Since the dysregulation of CDKs is a frequently occurring event during tumorigenesis, CDKs have evolved as important target proteins for cancer treatment. As a transcription-associated CDKs, the dysregulation of the *CDK12* gene has been recently reported in different types of malignancies and contributes to cancer progression and aggressiveness. The purpose of this study is to explore the exact functions and underlying mechanisms of *CDK12* for tumorigenesis and proliferation in cervical cancer.

The mutations, amplifications, deletions, or fusion of *CDK12* gene have been recently reported in different types of cancers, such as loss-of-function mutations of genomic are correlated with tandem duplications (TDs) and homologous recombination (HR) in high-grade serous ovarian carcinoma and prostate cancer, which suggests that *CDK12* is a tumor suppressor. On the contrary, the most common genomic alteration of *CDK12* is amplifications. Overexpression of *CDK12* has some properties of an oncogene in other tumors and promotes the proliferation of *HER2*-positive breast cancer, which results in metastasis and poor prognosis. In this study, widespread computational bioinformatic analysis from some independent databases and clinical patient tissue specimens show that the gene and protein level of CDK12 significantly increase in cervical cancer, and the overexpression of *CDK12* is closely correlated to the poor prognosis of patients suffering from cervical cancer. Therefore, we believe that *CDK12* may be a poor prognostic marker for cervical cancer.

It is known that the knockdown of *CDK12* can inhibit the progression of the cell cycle of esophageal and lung cancer [18, 19]. Previous studies found an accumulation peak of CDK12 in the early G1 phase, and it played a crucial role in the transition of G1 to the S phase [20]. Cell cycle analysis showed that knockdown of CDK12 reduced the progression of the G1/S phase, which was an essential process for DNA replication in the cell cycle [21]. Similarly, in the present study, we found that the knockdown of *CDK12* inhibited progression from G1 to S phase and DNA replication in cervical cancer cells. Suppression of *CDK12* or THZ531, CDK12 inhibitor, has been shown to inhibit cell proliferation and induce apoptosis in cancer cells [22]. In the present study, we also found that knockdown of *CDK12* with shRNA or THZ531 treatment inhibited cell proliferation, suppressed colony formation, and promoted their apoptosis in cervical cancer cells.

TAMs, as a kind of myeloid immune cell, have been reported to the important prognostic significances in human malignancies [16]. Current studies found that an increased infiltration in the TME is associated with a worse prognosis for lung and breast cancer patients [23, 24]. Besides lung and breast cancer, increased accumulation of TAMs in gastric cancers and multiple myeloma is also associated with a poor prognosis [25]. CDK12 contributes to regulating oncogenes and biological pathways, such as super-enhancer- (SE-) associated genes and NF-*κ*B pathway, which is involved in homeostatic control of the immune system [26]. Meanwhile, loss-of-function of *CDK12* is a common correlation with TD phenotype and increases T lymphocyte infiltration, leading to sensitization of cancer cells to some immune checkpoint inhibitors, such as antiprogrammed cell death-1 (PD-1) in prostate and ovarian cancers [27, 28]. In the present study, Gene-Set Enrichment Analysis in cervical cancer cells revealed that the altered expression of genes was enriched in the NF-*κ*B pathway and immune-inflammatory response pathways. Moreover, the overexpression of *CDK12* enhances the TAMs infiltration by immunohistochemistry techniques and gene expression profiling. These results indicated that CDK12 may be contributed to the regulation of TME in CC cells.

*CDK12*, which is a transcription-associated CDKs, is responsible for gene transcription initiation and elongation through the phosphorylation of the CTD of RNAP II at Ser2 and Ser5. *CDK12* also regulates the expression of DDR and DNA replication genes, which are involved in cell cycle and genomic stability, by participating in RNA splicing and translation [29]. CDK12 is a macromolecular substance, which comprises 1490 amino acids with a mass of 164 kDa [30]. To ensure accurate gene transcription-associated function of CDK12, the nuclear-cytoplasmic transport of CDK12 protein requires karyopherin*β* protein (Kap*β*s) mediation. In the present study, our western blotting and immunofluorescence results first revealed that a new molecular mechanism for nuclear-cytoplasmic transport of CDK12, which was modulated by TNPO1. TNPO1 mediated the nuclear import of CDK12 and then ensured CDK12 to exert its function of regulating gene transcription.

## 5. Conclusion

In the present study, our results showed that overexpression of CDK12 was associated with CC progression and poor prognosis. Knockdown of CDK12 inhibits the cell cycle process and proliferation in cervical cancer cells. The GSEA analysis results provide insight into the roles of CDK12 in immune microenvironment modulation. We also found that the nuclear import of CDK12 was mediated by TNPO1. These findings strongly suggest that CDK12 is a potential immunotherapy target in cervical cancer.

## Data Availability

The data used to support the results of this study were supplied by Yincheng Teng under license and so cannot be made freely available. Requests for access to these data should be considered by the corresponding author.

## Supplementary Materials

Supplementary Materials Supplementary Figure 1 showed the level of expression of *CDK12* mRNA in CC cell lines and interfere or overexpression efficacy of CDK12.
