# Supplementary material for: CDK12 Promotes Cervical Cancer Progression through Enhancing Macrophage Infiltration
Source: J Immunol Res. 2021 Feb 11;2021:6645885. doi: 10.1155/2021/6645885 (PMC7892235; doi:10.1155/2021/6645885)
Supplement: Supplementary Materials — Supplementary Figure 1 showed the level of expression of CDK12 mRNA in CC cell lines and interfere or overexpression efficacy of CDK12. [file 6645885.f1.pdf]

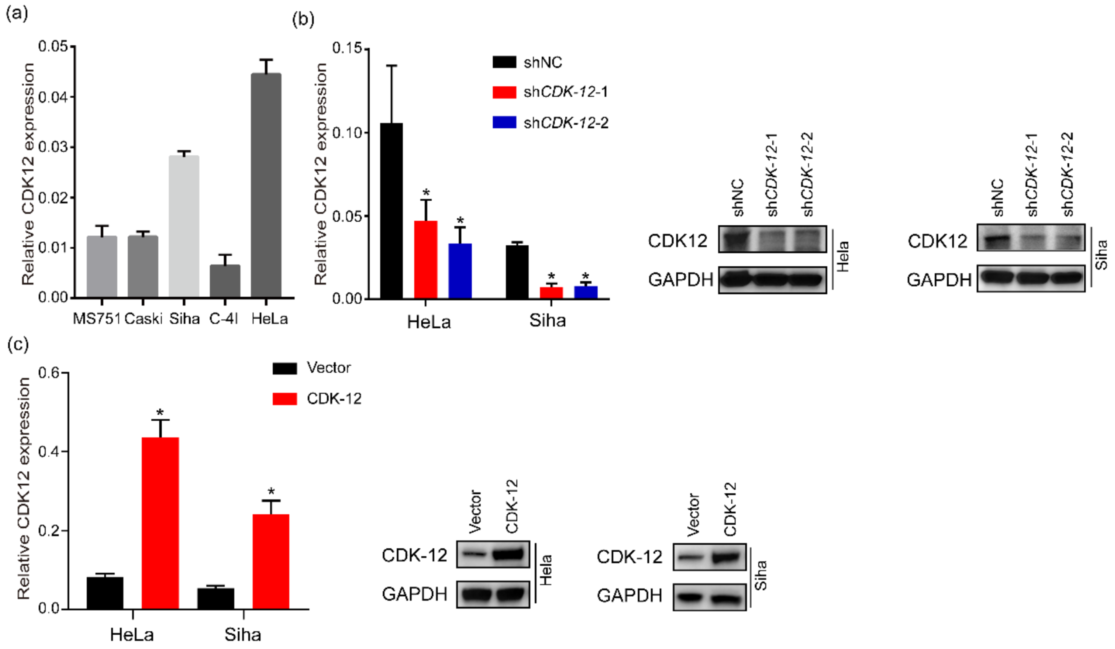

Supplementary Figure 1 (a) The expression of *CDK12* mRNA was detected by real-time quantitative PCR in CC cell lines. (b) Interfere efficacy of *CDK12* in Hela and Siha cells with either shRNA (sh *CDK12*-1, sh*CDK12*-2) or shNC were analyzed by qRT-PCR and western blotting. (c) Over-expression efficacy of *CDK12* in Hela and Siha cells with CDK12 or vector were analyzed by qRT-PCR and western blotting.
